# Regenerative Microbiology: Harnessing Bacterial Antagonism and Spatiotemporal Signaling for Diabetic Wound Repair

**DOI:** 10.1002/smmd.70044

**Published:** 2026-08-12

**Authors:** Sabri Saeed Sanabani

**Affiliations:** ^1^ Laboratory of Medical Investigation (LIM‐03) Hospital das Clínicas (HCFMU), Faculty of Medicine, University of São Paulo São Paulo Brazil; ^2^ Laboratory of Medical Investigation in Dermatology and Immunodeficiencies (LIM‐56) Department of Dermatology Faculdade de Medicina Instituto de Medicina Tropical de SP Universidade de São Paulo São Paulo Brazil

**Keywords:** bacteriophages, diabetic wounds, dysbiosis, microbial antagonism, microbial ecology, microbiome therapy, probiotics, tissue regeneration

## Abstract

Chronic diabetic foot ulcers represent a persistent clinical challenge characterized by a “locked” inflammatory phase, biofilm‐mediated infection, and impaired tissue regeneration. Because conventional antibiotic and debridement therapies fail to resolve dysbiosis or stimulate healing, microbial antagonism has emerged as a potent biological principle for wound restoration. This narrative review integrates ecological evidence and mechanistic insights from animal models and early human studies to explore how beneficial microorganisms, including probiotics, bacteriophages, and competitive consortia, actively reshape diabetic wound environments. We examined the multifaceted mechanisms of these interactions, ranging from direct pathogen inhibition to host immunomodulation and metabolic signaling via conserved pathways such as the p40/epidermal growth factor receptor (EGFR)/PI3K axis. To address significant translational barriers, we introduce a “Regenerative Microbiology” framework that emphasizes spatiotemporal coordination and precision stratification. By tailoring the use of live biotherapeutics or metabolically independent postbiotics to a patient's vascular and microbial profiles, this approach offers a strategic roadmap for transforming the management of infection‐driven tissue damage in chronic diseases.

## Key Points

- Introduction of the “Regenerative Microbiology” framework: This manuscript introduces a clinical framework that applies spatiotemporal coordination and precision medicine to address the “locked” inflammatory arrest and impaired tissue regeneration observed in chronic diabetic foot ulcers (DFUs).
- Shift to ecological biointerventions: Conventional antibiotic and debridement therapies often fail to resolve dysbiosis or address underlying cellular senescence. Using “microbes against microbes” via bacterial antagonism provides a biological approach to restore wound ecology and promote healing.
- Selective predation and biofilm disruption: Bacteriophage therapy offers a highly specific predation mechanism that targets and lyses multidrug‐resistant pathogens within persistent biofilms while preserving the beneficial commensal microbiota.
- Reprogramming host immune responses: Beneficial microorganisms facilitate the transition from a pro‐inflammatory M1 state to a reparative M2 macrophage phenotype, primarily by activating conserved host signaling pathways such as the p40/EGFR/PI3K axis.
- Precision stratification by vascular profile: A strategic clinical roadmap uses vascular health assessments (Ankle‐Brachial Index) to determine the optimal intervention, recommending live biotherapeutics for perfused wounds and “metabolically independent” postbiotics or extracellular vesicles for ischemic conditions.

## Graphical abstract

This review introduces “Regenerative Microbiology,” a framework that uses bacterial antagonism and phage therapy to address diabetic wound dysbiosis. By activating the p40/EGFR/PI3K axis and providing a vascular‐stratified precision roadmap, this approach shifts the paradigm from antimicrobial suppression to intelligent, microbe‐driven tissue restoration.

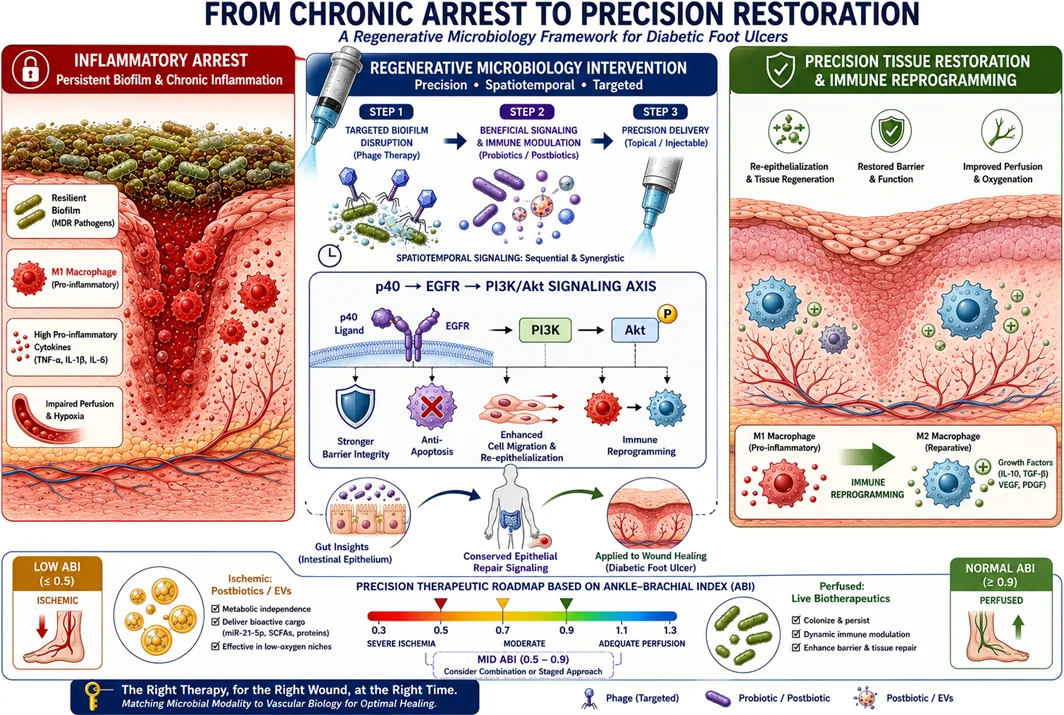

## 1 Introduction

The global prevalence of diabetes mellitus is a growing health crisis, affecting approximately 537 million adults as of 2021. This number is projected to increase to 783 million by 2045 [1]. The most serious complication is the development of chronic wounds, particularly diabetic foot ulcers (DFUs), which have a lifetime incidence risk of 19%–34% in patients with diabetes [2]. These wounds represent a significant human and economic burden, as global diabetes‐related healthcare spending is expected to reach $1.054 trillion by 2045 [3]. The natural history of these ulcers is sobering; they precede approximately 85% of all diabetes‐related lower extremity amputations, with a limb lost somewhere in the world every 30 s [4]. Furthermore, the mortality rate for patients with DFUs is 2.5 times higher than for those without ulcers, and the 5‐year mortality rate after amputation may exceed 70% [5].

This review was developed through a comprehensive synthesis of the literature indexed in PubMed, Scopus, and Web of Science, covering the period from 2000 to 2026. The search focused on keywords including “bacterial antagonism,” “diabetic foot ulcer,” “bacteriophage therapy,” “probiotics,” and “wound healing.” The selection criteria prioritized high‐quality mechanistic studies, clinical trials, and recent taxonomic reclassifications to provide an up‐to‐date overview of microbe‐driven tissue repair. Where appropriate, author‐developed conceptual figures are included to integrate current evidence, illustrate proposed translational strategies, and highlight areas requiring further experimental and clinical validation.

### 1.1 Burden and Pathophysiology of Diabetic Wounds

**FIGURE 1:**
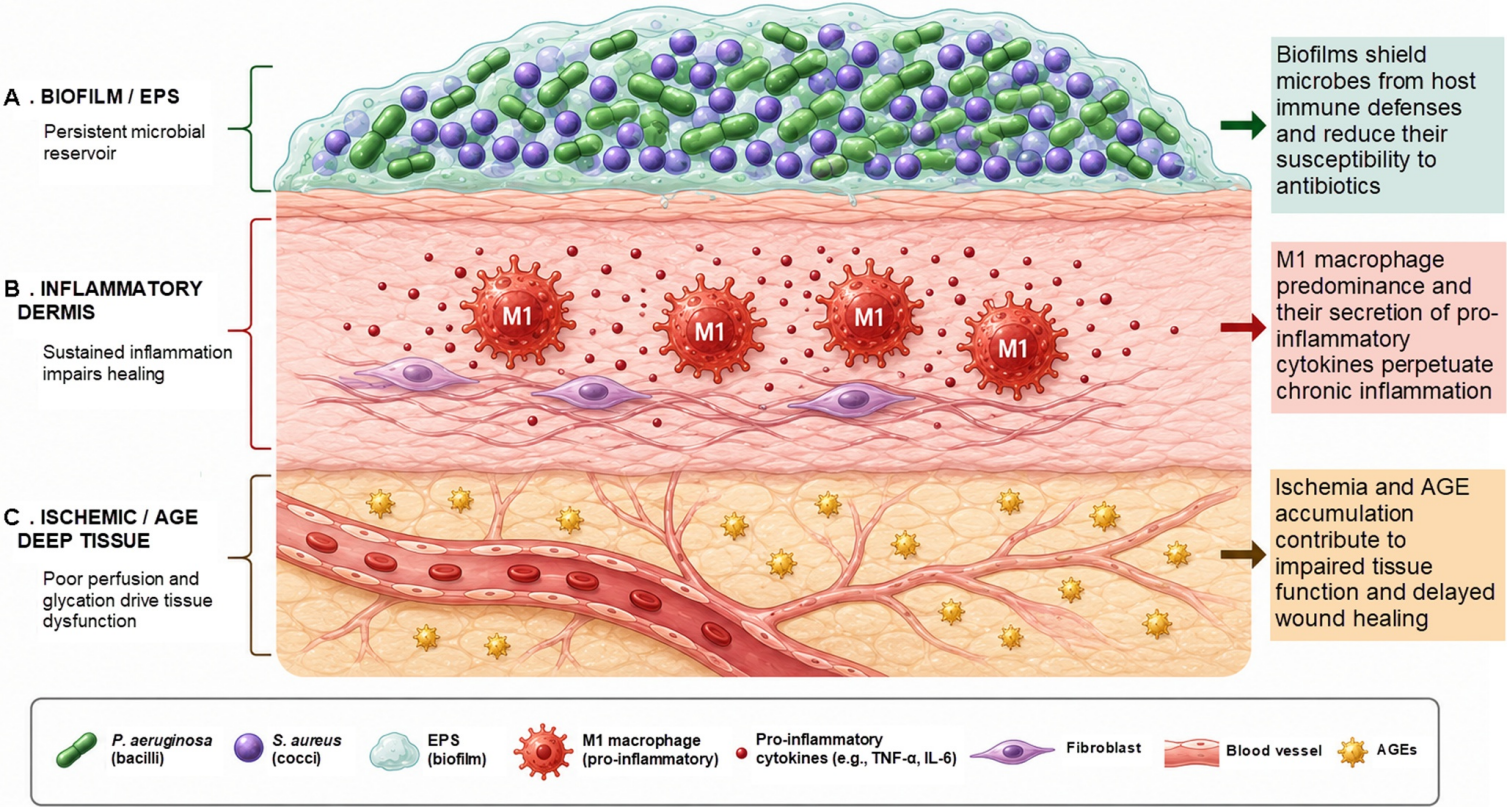
The biofilm fortress and host‐immune arrest in the diabetic wound microenvironment. This cross‐sectional illustration depicts the biological barriers to healing in chronic diabetic ulcers. (A) Biofilm/EPS layer: planktonic pathogens transition into a dense, surface‐attached biofilm encased within a protective extracellular polymeric substance (EPS) matrix. (B) Inflammatory dermis: a cluster of iNOS+ M1 macrophages represents the characteristic “stalled” inflammatory phase. (C) Ischemic/AGE deep tissue: constricted vasculature and the accumulation of advanced glycation end‐products (AGEs) create a hostile environment that inhibits cellular proliferation, tissue repair, and antimicrobial efficacy.

The pathophysiology of these wounds involves a complex interplay of metabolic and vascular dysfunctions that disrupts the normal sequence of tissue repair [2]. Chronic hyperglycemia triggers oxidative stress and activates biochemical pathways, including the polyol pathway and the advanced glycation end‐product (AGEs) pathway, which cause endothelial dysfunction and microvascular damage [6, 7]. Vascular impairment, often combined with peripheral neuropathy and ischemia, leads to persistent inflammation and localized hypoxia [8]. In this environment, wounds typically stall during the transition from the inflammatory to proliferative phase [9]. This arrest is characterized by impaired macrophage polarization, where failure to shift from the pro‐inflammatory M1 phenotype to the reparative M2 phenotype prevents proper angiogenesis, granulation, and re‐epithelialization [7] (Figure 1B: Inflammatory dermis). This is further complicated by the high prevalence of polymicrobial biofilms, present in up to 60%–80% of chronic wounds, which provide physical and metabolic barriers to pathogens, such as *Staphylococcus aureus* and *Pseudomonas aeruginosa* [10] (Figure 1A: Biofilm/EPS).

### 1.2 Limitations of Current Antimicrobial and Wound‐Care Approaches

Current antimicrobial and wound care approaches often fail to resolve these complex infections or restore the microbial balance [7]. Conventional management includes surgical debridement, pressure offloading, and systemic or topical antibiotics [8]. However, these methods often fail to adequately penetrate the extracellular polymeric substances (EPS) of biofilms, which can be up to 1000 times more resistant to antibiotics than planktonic cells [11]. The escalating threat of antimicrobial resistance (AMR), particularly among “ESKAPE” pathogens such as MRSA and multidrug‐resistant (MDR) *Pseudomonas aeruginosa*, further limits their therapeutic efficacy [11]. Moreover, prolonged antibiotic use can disrupt the gut and skin microbiota, potentially destabilizing the host's natural defenses [12, 13]. Traditional antibiotics lack pro‐regenerative effects and do not address the underlying cellular senescence or impaired growth factor signaling that maintain diabetic wounds in a chronic state [14].

### 1.3 Concept of “Microbes Against Microbes”

These limitations have made it necessary to explore new paradigms, such as the concept of “microbes against microbes,” which harness natural ecological principles to restore health. Microbial antagonism uses beneficial microorganisms to compete with pathogens for nutrients and adhesion sites and to secrete antimicrobial substances such as bacteriocins, hydrogen peroxide, and organic acids [3, 15]. Innovative strategies also target quorum‐sensing interference, disrupting the cell‐to‐cell communication that pathogens use to coordinate biofilm formation and express virulence [11, 16]. Furthermore, bacteriophage therapy provides a highly specific predation mechanism in which viruses target and lyse‐specific bacterial hosts while leaving beneficial commensal microbiota intact [17, 18, 19]. This review examines how antagonistic microbes, including probiotics, engineered consortia, and phages, can be used to control persistent infections, modulate the host immune response, and stimulate tissue repair in a challenging diabetic microenvironment.

## 2 Microbial Ecology and Dysbiosis of the Chronic Diabetic Wound Ecosystem

The chronic wound microbiome functions as a dynamic ecosystem characterized by significant bacterial colonization and persistent pathological inflammation [20, 21]. Typical taxa identified in DFUs include *Staphylococcus aureus*, detected in up to 94% of some patient cohorts, *Pseudomonas aeruginosa*, various *streptococci*, *Escherichia coli*, and opportunistic pathogens, such as *Enterococcus faecalis* [22, 23]. This polymicrobial consortium often includes anaerobic bacteria, such as *Bacteroides* and *Prevotella*, along with fungal communities dominated by *Candida albicans*, all of which coexist within complex, structured polymicrobial biofilms [24, 25, 26]. These biofilms serve as physical and metabolic fortresses, using an EPS matrix to shield microorganisms from host immune recognition and reduce antibiotic penetration by up to 1000 times compared to planktonic cells [27]. Within this spatial structure, microbial populations coordinate their virulence and antibiotic tolerance through quorum sensing, which is a cell‐to‐cell communication system that synchronizes the expression of toxins and proteases once the bacterial population reaches a threshold density [28].

This environment represents profound dysbiosis compared to intact skin. Healthy skin is characterized by high microbial diversity and protective commensals such as *Staphylococcus epidermidis*, whereas DFU microbiota exhibit significantly reduced species richness and evenness [29, 30]. Furthermore, the temporal stability of the microbiome is a critical predictor of healing outcomes, as stable pathogenic community types are often associated with poor healing trajectories and prolonged wound duration [31]. The survival and virulence of these microbial communities depend on complex interactions, ranging from synergistic cooperation between *Staphylococcus aureus* and *Pseudomonas aeruginosa*, which enhances their shared antibiotic tolerance and increases the secretion of tissue‐destructive enzymes, to antagonistic relationships in which microbes compete for limited nutrients and adhesion sites [32]. These pathogens organize into recalcitrant structures that shield bacteria from host immunity and antibiotic penetration (Figure 1).

Beneficial microorganisms can improve the wound microenvironment by producing inhibitory metabolites such as bacteriocins, hydrogen peroxide, and organic acids, which lower the local pH and disrupt the quorum‐sensing signals used by pathogens [7]. The local microenvironment of a diabetic foot ulcer creates a harsh environment that directly affects the viability of therapeutic probiotics. Healthy adult skin typically maintains a mildly acidic surface pH of approximately 4.5–5.0 (the “acid mantle”), which supports resident commensal flora and limits colonization and spread of pathogenic bacteria [33]. In contrast, chronic diabetic wounds often have a relatively alkaline environment, with reported pH values ranging from about 7.4 to 8.9 [34]. This alkalinization can disrupt the proton motive force and membrane transport processes of probiotic strains not adapted to high external pH. Persistent hyperglycemia in chronic diabetic wounds also leads to elevated glucose and other solute concentrations in wound exudate, increasing local osmolarity in the tissue microenvironment [35, 36]. Hyperosmotic conditions promote water efflux and cellular dehydration, which can suppress metabolic activity and stress tolerance in exposed microbial cells. Therefore, selecting or engineering probiotic strains that tolerate both high‐glucose and hyperosmotic conditions and alkaline pH is essential to ensure robust local viability in the diabetic wound microenvironment.

In addition to these environmental factors, evaluation of systemic safety remains a high priority. In patients with advanced diabetes who may be severely immunocompromised or have compromised vascular barriers, there is a small but critical risk that topically applied live bacteria can enter the bloodstream, potentially causing bacteremia or systemic sepsis. This risk underscores the need for strict screening protocols that prioritize strains with minimal translocation risk, documented genomic safety, and absence of transmissible antibiotic resistance genes [37, 38].

These complex ecological dynamics make diabetic wounds an ideal model system for therapeutically exploiting microbial antagonism in the clinical settings [39, 40, 41]. Furthermore, they provide measurable outcomes for monitoring therapeutic success, including quantifiable weekly surface area reduction, specific histological markers of angiogenesis and collagen deposition, and indicators of infection resolution [7]. This combination of rich polymicrobial ecology and highly visible, quantifiable healing phases results in diabetic wounds an attractive model for evaluating microbiome‐based interventions [15, 42].

## 3 Mechanisms of Bacterial Antagonism: Microbes Against Microbes

### 3.1 Core Antagonistic Mechanisms

**FIGURE 2:**
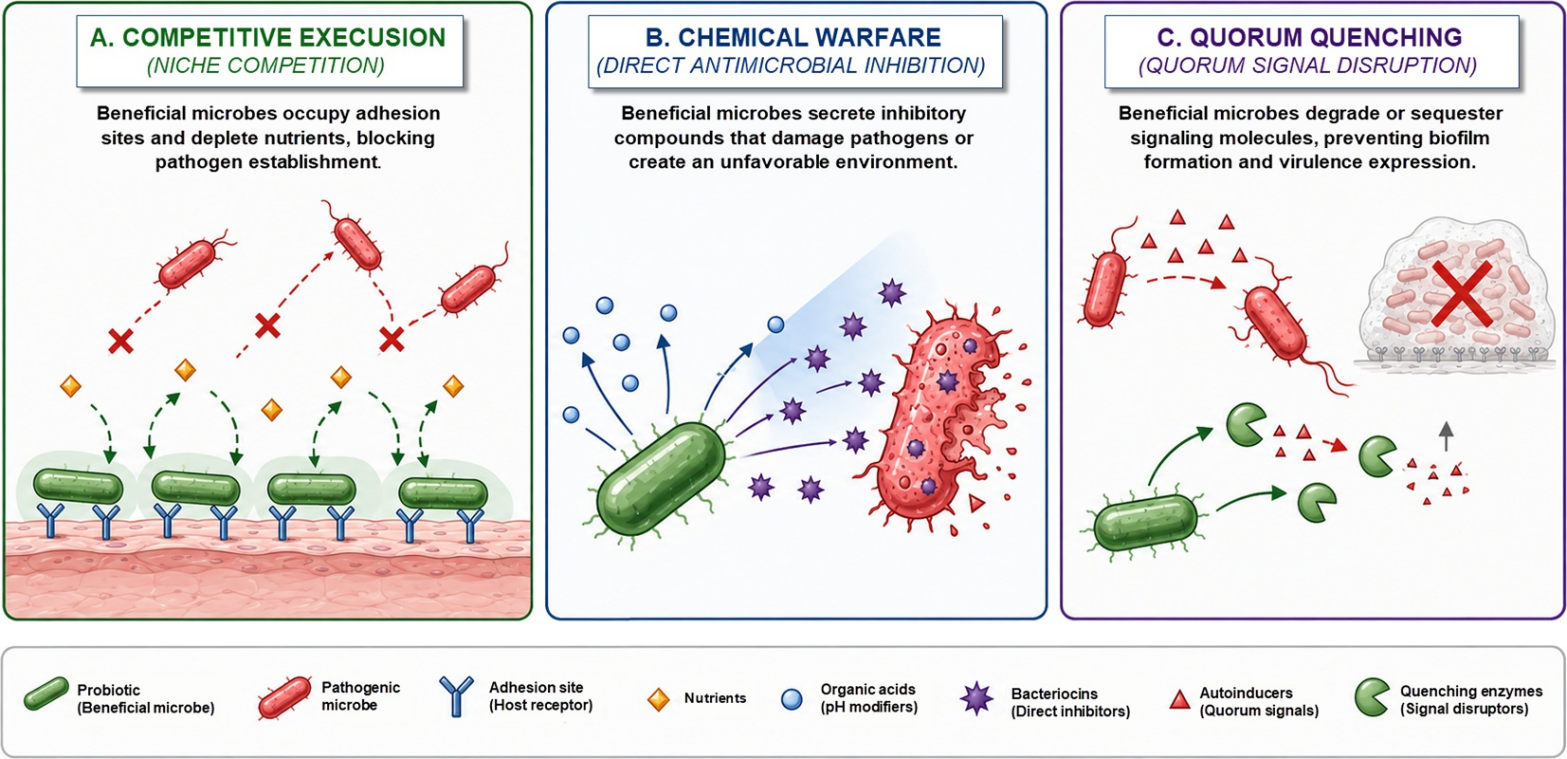
Molecular mechanisms of bacterial antagonism in the wound milieu. (A) Competitive exclusion: Beneficial microbes (green) physically obstruct pathogenic adherence by occupying specific adhesion receptors and outcompeting pathogens for limited essential nutrients. (B) Chemical warfare: The secretion of organic acids (e.g., lactic acid) induces a localized pH shift, while the production of specialized bacteriocins facilitates direct cell wall lysis and membrane disruption in target pathogens. (C) Quorum quenching: Probiotic‐derived enzymes, such as lactonases, degrade pathogenic autoinducer molecules (triangles). This disruption of quorum sensing prevents the coordinated gene expression required for biofilm maturation and virulence factor production.

The core of microbial antagonism in chronic wounds is based on the ecological principle of competitive exclusion, where commensal and probiotic microorganisms occupy epithelial binding sites and consume limited nutrients, thereby restricting the ability of pathogenic species to colonize and expand [42]. On the skin and mucosal surfaces, *Staphylococcus epidermidis* exemplifies colonization resistance, as experimental models have shown that precolonization or inoculation with *Staphylococcus epidermidis* can prevent or reduce subsequent *Staphylococcus aureus* or MRSA colonization, partly by competing for adhesion sites and niches [43, 44, 45]. Similarly, probiotics applied to skin or wounds inhibit various pathogens through competitive exclusion in vitro and in vivo, reducing wound infection rates in animal models and clinical cohorts [46]. These antagonistic microbes use a diverse chemical arsenal to suppress competitors, including organic acids such as lactic acid, which lowers the local pH and inhibits the growth of pathogens [47, 48] (Figure 2B: Chemical warfare). Furthermore, many antagonistic commensals and probiotics secrete potent antimicrobials, such as bacteriocins (e.g., nisin from *Lactococcus lactis* and epidermin from *staphylococcal species*) and lipid‐active peptides, such as phenol‐soluble modulins from *Staphylococcus epidermidis*, which selectively kill competing pathogens while sparing producers [49, 50]. These strains also produce hydrogen peroxide and other reactive oxygen species (ROS), which contribute to peroxidase‐mediated suppression of pathogens [51]. Beyond direct killing, probiotics can use more advanced interference strategies, including quorum‐sensing disruption and quorum quenching, in which lactic acid bacteria (LAB) release metabolites or enzymes that hijack or degrade autoinducer signals in LuxRI‐, Agr‐, or AI‐2/LuxS‐type systems, thereby blocking biofilm formation and virulence programs of pathogens without affecting their growth [52, 53] (Figure 2C: Quorum quenching). Complementing these strategies is bacteriophage‐mediated killing, which provides a highly specific predation mechanism that selectively lyses pathogenic bacteria, including MDR strains, while largely sparing the surrounding commensal flora [54]. Importantly, many therapeutic phages and their associated depolymerases can penetrate or disrupt complex biofilm matrices, reduce bacterial load in chronic wound biofilms, and enhance the efficacy of concurrent antibiotics and debridement [2, 55]. Antagonistic microbes use a multifaceted arsenal ranging from physical niche competition to biochemical interference (Figure 2).

Microbial competitive exclusion operates as a principal defense mechanism within the wound bed, where therapeutic strains outcompete deep‐seated pathogens for localized physical niches and essential micronutrients. As illustrated schematically in Figure 2A, this spatial segregation restricts pathogenic attachment to the extracellular matrix. This process is driven by the local secretion of high‐affinity siderophores that deplete available iron, along with the occupation of cell‐surface receptors by probiotic surface proteins, which effectively halts pathogen colonization through direct steric hindrance.

### 3.2 Antagonism and Host Modulation

Beyond direct microbial warfare, these antagonistic microbes actively modulate host defenses to transform the wound environment, induce antimicrobial peptides (AMPs) and immunoglobulins, reprogram innate and adaptive responses, and promote re‐epithelialization and neovascularization in experimental and clinical settings [56, 57]. Beneficial commensals, such as *Staphylococcus epidermidis*, interact with host immune cells to induce the expression of AMPs in keratinocytes, including human *β*‐defensin‐3 and related *β*‐defensins, thereby reinforcing the innate barrier of the skin. *Staphylococcal* stimulation of keratinocytes also increases cathelicidin (LL‐37) transcripts, further contributing to antimicrobial protection at the wound surface [58, 59, 60]. This interaction can “train” the local immune system, as commensal‐specific T‐cells induced by the skin microbiota have been shown to couple antimicrobial effector functions with transcriptional programs that accelerate wound closure. These T‐cells serve as tissue sentinels that rapidly shift to produce tissue‐repair cytokines after injury. Keratinocytes amplify these responses through the TLR‐dependent sensing of commensal signals by producing cytokines, chemokines, and AMPs that promote re‐epithelialization and barrier restoration [61, 62]. A critical aspect of immunomodulation is its effect on macrophage polarization. Probiotic signals, such as extracellular vesicles (EVs) derived from *Lactiplantibacillus plantarum*, that drive macrophages toward an anti‐inflammatory M2 phenotype in human skin, and nisin‐loaded microneedle systems that shift macrophages from iNOS^+^ M1 to CD206^+^ M2 in infected diabetic ulcers, help transition persistent, destructive inflammation to a reparative, pro‐healing state [63, 64, 65, 66]. Additionally, microbial metabolites such as short‐chain fatty acids (SCFAs) have significant immunoregulatory effects. In particular, butyrate and related SCFAs promote the differentiation and expansion of Foxp3^+^ regulatory T‐cells and enhance their IL‐10–mediated suppressive capacity, thereby reducing excessive inflammation and limiting collateral tissue damage [67, 68].

### 3.3 Concept of “Regenerative Microbiology”

This dual functionality supports a “regenerative microbiology” framework, in which selected microbes or microbial products can both limit the pathogen burden and promote repair‐associated signaling. Microbiota‐derived indoles and related tryptophan metabolites have been associated with anti‐inflammatory effects, epithelial barrier support, and wound repair processes including enhanced epithelial migration and other regeneration‐associated cellular responses [69]. In addition, EVs derived from the probiotic *Lacticaseibacillus rhamnosus GG* have been shown to accelerate skin wound healing, promote re‐epithelialization and angiogenesis, and involve miR‐21‐5p–dependent signaling pathways [70]. Restoring the microbial ecological balance in chronic diabetic wounds may help reestablish the metabolic and immune signals needed for tissue regeneration [71].

## 4 Probiotics and Competitive Bacterial Consortia in Diabetic Wounds

Animal studies have shown that topical probiotics and probiotic‐derived consortia can improve diabetic wound healing by reshaping the wound microenvironment, reducing the pathogen burden, and promoting tissue regeneration. In diabetic rodent models, strains such as *Lactiplantibacillus plantarum*, *Lacticaseibacillus rhamnosus GG*, *Lactobacillus acidophilus*, and *Bifidobacterium animalis* subsp. *lactis BB‐12* are associated with faster wound closure, reduced inflammation, improved granulation tissue, increased collagen deposition, and enhanced neovascularization [71, 72]. These findings support the broader concept that probiotic microbes help repair barriers and restore the microbial balance in chronic wounds.

### 4.1 Evidence From Animal Model

Evidence from diabetic wound models indicates that probiotics act through both antimicrobial and host directed repair mechanisms. In an infected rat wound model, topical *L*. *rhamnosus GG* and *BB‐12* accelerated healing, increased granulation tissue and collagen deposition, and reduced pathogenic bacteria such as *Staphylococcus* and *Proteus*. These clinical improvements were accompanied by a shift in the local ecosystem toward beneficial taxa, including *Corynebacterium*, alongside reduced neutrophil infiltration and modulation of inflammatory mediators, changes that are consistent with a transition from a persistent inflammatory state to tissue repair [71]. Furthermore, viable *lactobacilli* formulations, particularly *Lactobacillus acidophilus* and *Lacticaseibacillus rhamnosus*, have significant effects on collagen synthesis, fibroblast proliferation, and angiogenesis [73, 74]. This suggests that distinct strains may preferentially influence specific phases of wound repair, from initial pathogen clearance to late‐stage maturation of the extracellular matrix (ECM) [75, 76].

### 4.2 Competitive Consortia

The concept of competitive bacterial consortia is particularly relevant in chronic diabetic wounds, which are often polymicrobial. Instead of relying solely on the pharmacological suppression of pathogens, beneficial microbes may promote healing by outcompeting opportunistic bacteria and restoring microbial balance [77]. In this context, probiotics reduce colonization by *Staphylococcus aureus* through nutrient competition and secretion of antimicrobial metabolites such as bacteriocins and organic acids [78, 79].

Beyond infection control, these consortia actively support tissue regeneration by modulating macrophage polarization toward a reparative M2 phenotype and increasing the expression of pro‐healing factors, such as vascular endothelial growth factor (VEGF) and transforming growth factor *β* (TGF‐β) [56, 70, 80]. Innovative delivery systems, such as probiotic‐loaded hydrogels, have further enhanced these effects by providing sustained action against biofilms [81, 82].

### 4.3 Human Studies in DFUs

Current human studies on probiotics for DFUs show a significant shift toward microbiome‐based adjuncts to standard care, with clinical trials exploring both topical and oral administrations. A notable randomized controlled trial (*n* = 22) conducted in Argentina in 2022 investigated the efficacy of weekly topical *Lactiplantibacillus plantarum* for 12 weeks following standard surgical debridement [83]. The findings from this trial showed that patients receiving probiotics had greater wound area reduction (73.5%) than those receiving debridement alone (45.8%) and higher overall closure rates. Similarly, a multicenter retrospective study in Taiwan using a probiotic soybean‐based concentrate reported that 83% of non‐infected DFUs achieved complete closure within 16 weeks, often with significant reductions or clearance of persistent gram‐negative pathogens such as *Proteus mirabilis* and *Klebsiella pneumoniae* [84]. Case reports have further supported these outcomes, demonstrating that multi‐strain probiotic formulations containing *Lactobacillus acidophilus*, *Lactiplantibacillus plantarum*, and *Streptococcus thermophilus* are effective.

The choice between systemic and topical probiotic interventions remains an active area of clinical research. Recent evidence indicates that systemic probiotic administration can improve metabolic marker levels. A systematic review and meta‐analysis by Memon et al. [85] found that combining probiotics with metformin improved glycemic and metabolic outcomes in type 2 diabetes, while Yang et al. [86] developed microfluidic microspheres for the co‐delivery of probiotics and postbiotics. Adapting these microfluidic microsphere delivery systems for topical application could help maintain high probiotic viability in the wound bed, protect therapeutic strains from local environmental stresses, and ensure controlled release.

Key findings on infection control indicate that these interventions significantly reduce the bacterial counts and biofilm burden in complicated DFUs [87]. Probiotics achieve this through microbial antagonism, secreting organic acids and bacteriocins that lower the local pH and are believed to disrupt the quorum‐sensing signals of pathogens used to coordinate biofilm stability and virulence [88]. In addition to antimicrobial effects, histological assessments in clinical cohorts have shown that probiotic application significantly improves angiogenesis and fibroplasia [83]. This is characterized by increased neovascularization and vessel density as well as enhanced mature collagen deposition, all of which are essential for restoring tissue integrity in chronic, ischemic, and diabetic environments.

A critical immunomodulatory mechanism observed in these human studies is the ability of probiotic signals to drive macrophage polarization toward the reparative M2 phenotype [83, 89]. In chronic diabetic wounds, the healing process is typically stalled in a proinflammatory M1 state. The transition to M2 macrophages, facilitated by probiotics, enhances the phagocytosis of apoptotic cells and debris and triggers the release of key trophic factors such as VEGF and TGF‐β, which are associated with M2‐driven tissue repair [90, 91]. This modulation of the innate response transforms the wound from a site of persistent inflammation to active regeneration.

Furthermore, clinical research has identified systemic metabolic benefits associated with probiotic intervention in patients with DFU. In a double‐blind, randomized, placebo‐controlled trial of 60 patients with grade 3 DFUs, daily oral supplementation with a multi‐strain probiotic capsule for 12 weeks resulted in significant reductions in fasting plasma glucose, serum insulin, and HbA1c levels [92]. These systemic improvements in glycemic control were accompanied by a substantial increase in total antioxidant capacity and a reduction in high‐sensitivity C‐reactive protein and malondialdehyde levels, which are markers of oxidative stress. Collectively, these studies suggest that integrating probiotics into DFU management can provide dual benefits: directly accelerating local tissue repair and stabilizing the underlying systemic metabolic dysregulation that contributes to wound chronicity [7].

To maximize therapeutic efficacy, competitive consortia should be designed by selecting strains that target different phases of the wound healing process. Some taxa suppress pathogen‐driven inflammation, whereas others actively stimulate the proliferative and remodeling stages. The specific functional contributions of key LAB to these phases are summarized in Table 1.

**TABLE 1:** Phase‐specific contributions of probiotic strains to diabetic wound repair.

Healing phase	Primary biological action	Representative strains	Mechanistic insight	Representative evidence
Inflammatory	Pathogen exclusion & M1/M2 shift	*Lacticaseibacillus rhamnosus* GG, *Lactiplantibacillus plantarum*	Downregulation of pro‐inflammatory cytokines (TNF‐α, IL‐6) and biofilm disruption.	References [72, 80, 82, 83, 89]
Proliferative	Angiogenesis & Re‐epithelialization	*Limosilactobacillus reuteri*, *Lactobacillus acidophilus*	Stimulation of VEGF expression and acceleration of keratinocyte migration.	References [70, 83, 84, 93, 94, 95]
Remodeling	Collagen deposition & ECM integrity	*Lacticaseibacillus casei*, *Bifidobacterium longum*	Modulation of TGF‐β signaling and fibroblast‐to‐myofibroblast differentiation.	References [73, 83, 84, 90, 91, 95]

### 4.4 Advanced Mechanistic Insights Into Pathogen Antagonism

Although the fundamental principles of competitive exclusion are well‐documented, recent high‐resolution studies have quantified the strength of these interactions in diabetic wound environments. A key finding in recent research is the extent of biofilm disruption: specific probiotic‐derived metabolites have been shown to inhibit *Staphylococcus aureus* biofilm development by up to 95% [82].

Beyond direct competition, these consortia actively modulate the regenerative signaling and redox balance of the host. Certain strains enhance antioxidant defenses by upregulating enzymes, such as superoxide dismutase (SOD) and catalase, thereby reducing oxidative stress that impedes diabetic healing [96, 97]. The release of EVs enriched with microRNAs (e.g., miR‐21‐5p) directly stimulates keratinocyte proliferation and angiogenesis [70]. These interactions, along with a shift toward a reparative M2 macrophage phenotype, are associated with increased expression of VEGF and TGF‐β, accelerating the transition from the inflammatory phase to the proliferative phase of healing [70, 80].

The transition from a pro‐inflammatory to a reparative state is significantly influenced by microbial metabolites, particularly SCFAs such as butyrate. In addition to the miRNA‐mediated pathways previously discussed, butyrate acts as a signaling ligand for the G‐protein‐coupled receptor GPR109A on macrophage membranes. GPR109A activation suppresses NF‐κB signaling and promotes peroxisome proliferator‐activated receptor gamma (PPAR‐γ) expression. This metabolic reprogramming downregulates M1 markers, such as iNOS and TNF‐α, and upregulates M2‐associated genes, including CD206 and Arg‐1. In the diabetic wound bed, this shift is critical for resolving the persistent inflammatory phase and initiating SMAD‐dependent signaling required for TGF‐β activity and subsequent tissue remodeling.

### 4.5 Spatiotemporal Coordination of Interventions

**FIGURE 3:**
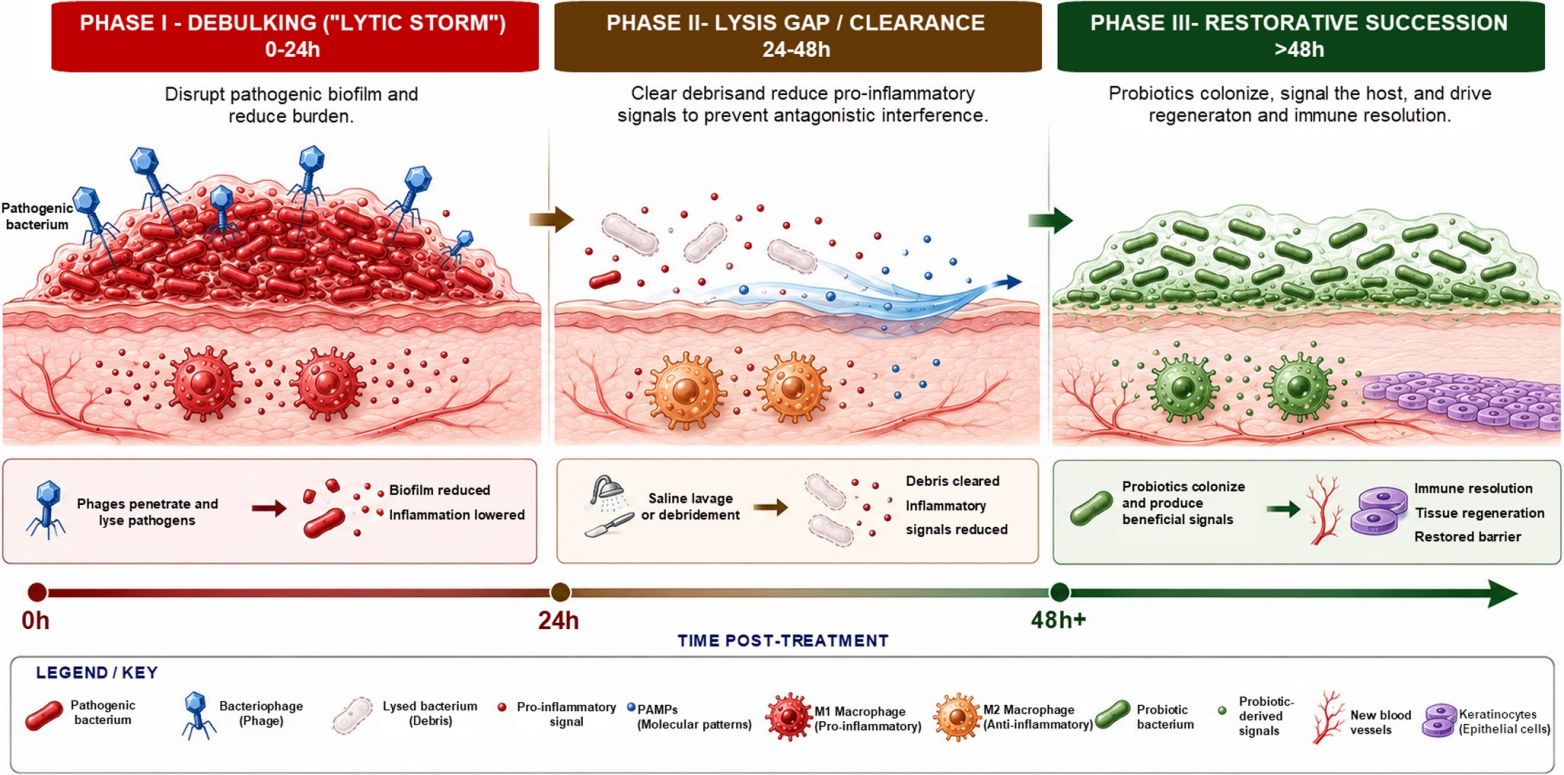
Proposed spatiotemporal succession framework for regenerative microbiology in diabetic wound repair. This timeline illustrates a sequential administration protocol designed to mitigate the “Double Duty” paradox and prevent antagonistic interference. Phase I (0–24 h): Lytic phages initiate rapid pathogen debulking and biofilm fragmentation. Phase II (24–48 h): The “Lysis Gap” facilitates the clearance of inflammatory debris and pathogen‐associated molecular patterns (PAMPs), restoring a receptive microenvironment. Phase III (> 48 h): Probiotic consortia (e.g., *Lp*. *plantarum*) colonize the cleared niche, secreting reparative signals, including p40, miR‐21‐5p‐enriched vesicles, and butyrate, to drive CD206^+^ M2 macrophage polarization, stimulate angiogenesis, and promote definitive re‐epithelialization. This figure represents a hypothesis‐generating conceptual framework synthesized from preclinical and emerging clinical evidence and should not be interpreted as a validated therapeutic protocol.

A key challenge in the ‘Regenerative Microbiology’ framework is the ‘Double Duty’ paradox: antagonistic interactions may occur between pathogen‐targeted phages and repair‐promoting probiotics. To minimize the risk of phages inadvertently lysing beneficial consortia through off‐target effects or shared surface receptors, we propose a sequential rather than concurrent administration strategy. To address this double‐duty paradox, we recommend a sequential succession model that separates lytic debulking from probiotic‐driven regeneration (Figure 3).

#### 4.5.1 Phase I: Phage‐Mediated ‘Debulking’

During the initial inflammatory phase, the primary objective is to disrupt established biofilms and reduce the pathogen load, such as *Staphylococcus aureus* or *Pseudomonas aeruginosa*. Lytic phages should be applied first to take advantage of their rapid replication kinetics, effectively clearing the niche for subsequent colonization (Figure 3, Phase I: Debulking).

#### 4.5.2 Phase II: Probiotic Recolonization and Tissue Repair

Probiotic consortia, such as *Lactiplantibacillus plantarum*, should be introduced only after the peak of the lytic cycle, typically 24–48 h after phage administration. This delay prevents premature competitive exclusion and ensures that probiotic strains promote M2 macrophage polarization and VEGF‐driven angiogenesis without interference from active viral predation or inflammatory debris resulting from rapid bacterial lysis (Figure 3, phase II: lysis gap and phase III: restorative succession). The specific parameters, rationales, and expected outcomes of this phased approach are summarized in Table 2.

**TABLE 2:** Phased spatiotemporal protocol for phage‐probiotic therapy in DFU repair.

Timing	Intervention	Rationale	Primary outcome
*T* = 0–24 h	Lytic phage cocktail	Rapid reduction of biofilm and pathogen density	Pathogen “debulking”
*T* = 24–48 h	Wound lavage/Debridement	Removal of lytic debris and PAMPs	Reduction of inflammatory “noise”
*T* > 48 h	Probiotic consortia	Niche occupation and promotion of angiogenesis/re‐epithelialization.	Regenerative healing

## 5 Bacteriophages and Phage–Bacteria–Host Triads in Diabetic Tissue Repair

### 5.1 Bacteriophage Therapy for DFU Infections

Bacteriophage therapy represents an innovative strategy to eliminate MDR pathogens in chronic diabetic wounds by using viruses that specifically target and lyse bacterial cells [98]. Clinical interest in this approach is driven by the rise in antibiotic resistance and failure of traditional regimens to resolve persistent infections in DFUs [99]. Evidence from preclinical models and early clinical investigations indicates that phages offer a high level of specificity, allowing them to eradicate specific pathogens without harming the beneficial commensal microbiota or human mammalian cells [42, 100]. For instance, topical phage cocktails have demonstrated superior performance compared to standard antibiotics, such as ceftriaxone, in promoting wound contraction and reducing bacterial loads in infected diabetic mouse models [12, 13]. Furthermore, unlike oral antibiotics, topical phage application does not disrupt the host intestinal microbiome, which is often a significant side effect of conventional systemic therapies [13].

The efficacy of phage therapy in resolving DFU infections is particularly notable because bacteriophages can multiply at the infection site and penetrate complex biofilm matrices [101]. Phages produce lytic enzymes and depolymerases that break down the physical barriers of biofilms, enabling the destruction of embedded bacteria, which are often 1000 times more resistant to antibiotics [102]. Additionally, specialized phages, such as ZCKP1, have been identified as potential treatments for MDR *Klebsiella pneumoniae* isolated directly from diabetic foot patients [103]. These interventions have shown promising outcomes, including significantly reduced bacterial colony counts and improved clinical healing when used alone or in combination with antibiotics such as gentamicin [100].

From cost‐effectiveness and safety perspectives, phage therapy offers distinct advantages over traditional pharmaceutical development [104]. Isolation and selection of new phages from nature is relatively rapid and less expensive than the years of research, and substantial financial investment is required to bring a new antibiotic to the market [105, 106]. Phages are also highly stable and can co‐evolve with their bacterial hosts to overcome emerging resistance, thus providing a more sustainable tool against ESKAPE pathogens [105]. Although the release of endotoxins during bacterial lysis remains a clinical concern that requires careful management, the overall safety profile of phages is supported by their long history of use in expanded access programs and lack of host cell toxicity [107, 108]. Therefore, bacteriophage therapy is emerging as a versatile and economical alternative to manage localized, treatment‐resistant infections in challenging diabetic microenvironments.

### 5.2 Mechanisms of Phage‐Mediated Antagonism

The mechanisms of bacteriophage‐mediated antagonism are primarily based on the ability of the viral life cycle to selectively target and destroy specific bacterial hosts without disrupting the broader microbial environment. The main mechanism is direct lysis, which begins when a phage recognizes a bacterial pathogen by matching its receptor‐binding proteins to specific receptors on the bacterial cell surface [109]. After binding, the phage injects its genetic material, hijacks the host's cellular machinery for replication, and ultimately induces bacterial cell lysis, releasing new phage particles to infect neighboring cells [110, 111]. This process is mediated by phage holins and endolysins, which allow endolysins to access the peptidoglycan layer and rapidly degrade the bacterial cell wall, leading to membrane permeabilization, host lysis, and release of progeny phages [112, 113].

A key advantage of phages in chronic diabetic wounds is their ability to penetrate the biofilms. The EPS matrix of a biofilm acts as a metabolic and physical barrier to antibiotics and immune cells, but phages can produce depolymerases and lytic enzymes that specifically break down these protective structural components [114, 115]. By degrading the matrix, phages access the embedded bacteria and physically disrupt the stability of the biofilm, potentially returning the infection to a treatable planktonic state [116]. Furthermore, phages show strong synergy with antibiotics, as demonstrated in wound healing models, where phage‐antibiotic combination therapy significantly improves bacterial clearance and wound contraction compared to monotherapy. In addition, phage‐antibiotic synergy has been demonstrated for gentamicin‐phage combinations, which enhance the killing of MDR bacteria beyond additive effects. Beyond simple synergy, engineered phages can re‐sensitize drug‐resistant pathogens to conventional antibiotics by delivering genetic cassettes that disrupt or replace resistance determinants, often using modified lysogenic or prophage‐like delivery systems [117, 118, 119, 120]. A major clinical challenge in treating chronic DFUs is the dense EPS matrix produced by polymicrobial biofilms, which acts as a physical barrier to conventional antibiotic therapy. Bacteriophages overcome this barrier by deploying specialized tail‐associated depolymerases, such as exopolysaccharides, alginate lyases, and glycoside hydrolases. These enzymes specifically target and cleave the complex carbohydrate and protein structures that form the EPS scaffold. By locally degrading this matrix, phages disrupt the structural integrity of the biofilm and create fluid channels that allow them to penetrate deeper layers of the wound [121, 122]. This degradation not only exposes deep‐seated pathogens to phage‐mediated lysis but also synergistically restores the efficacy of coadministered topical agents.

Unlike broad‐spectrum antibiotics, phage therapy can selectively target pathogenic bacteria while largely preserving the commensal microbiota, minimizing the disruption of microbial diversity [123]. By eliminating dominant pathogens in the dysbiotic DFU environment, phages open ecological niches and create favorable conditions for recolonization by beneficial commensals or the successful engraftment of applied probiotics [107]. This restorative shift is essential for transitioning the wound from a pathogen‐dense hyperinflammatory state to a balanced ecosystem that supports neovascularization and tissue regeneration [124].

### 5.3 Potential Impacts on Tissue Repair

The primary effect of bacteriophage therapy on tissue repair is achieved by effectively reducing the localized pathogen load and disrupting the biofilm burden, thereby breaking the cycle of chronic inflammation. By producing specialized depolymerases and lytic enzymes, phages degrade the EPS matrix, enabling the destruction of embedded bacteria, which typically obstructs healing [125]. Early evidence from diabetic mouse models showed that topical phage cocktails significantly reduce bacterial colony counts and accelerate wound contraction compared with standard antibiotic treatments [126, 127]. This clearance of infection directly affects molecular markers of regeneration by increasing the expression of proliferating cell nuclear antigen (PCNA), collagen (Col1a1), and fibronectin (Fn1), while simultaneously downregulating tissue‐destructive enzymes, such as MMP‐9 [119, 128]. By resolving the hyperinflammatory storm, phages create a favorable microenvironment that enables the initiation of granulation and angiogenesis, driven by the restoration of essential growth factor signaling such as TGF‐β [105, 115, 129].

In addition to directly killing bacteria, bacteriophages indirectly modulate host immunity through complex phage–immune interactions within the wound bed. Phages influence the signaling pathways of keratinocytes and macrophages, promoting the transition to a reparative state by altering the local cytokine profile [130, 131]. Successful therapy is characterized by a significant reduction in pro‐inflammatory mediators such as NF‐κB, TNF‐α, IL‐1β, and IL‐8, along with a measurable increase in anti‐inflammatory cytokines, such as IL‐10 and IL‐4 [131, 132]. However, a critical aspect of this interaction is the release of endotoxins and cellular debris during rapid bacterial lysis, which can trigger localized immune responses or, in cases of rapid systemic administration, severe inflammatory events such as the Jarisch‐Herxheimer reaction or cytokine storm [133, 134, 135]. In the context of DFUs, direct topical application remains the preferred route because it targets the infection site with minimal systemic exposure, localizes the immune response, and avoids broader metabolic or inflammatory complications [115, 136].

Conceptually, bacteriophages serve as precise ecological tools that offer a distinct advantage over broad‐spectrum antibiotics by preserving the integrity of the host microbiome [123, 137]. While systemic antibiotics often disrupt the microbiota and cause dysbiosis, significantly reducing both *α* and *β* diversity in the gut and on the skin, topical phage therapy is highly host‐specific and leaves the beneficial commensal community intact [138, 139, 140]. This specificity prevents secondary infections and the extensive ecological destabilization, which often follow conventional therapy. Furthermore, because phages reproduce only in the presence of their target bacterial hosts, they provide a self‐amplifying dose at the site of infection that naturally dissipates once the pathogen is eliminated [138, 141]. This evolutionary adaptability enables phages to co‐evolve with their bacterial hosts, overcoming emerging resistance in a manner that static antibiotic compounds cannot [42]. The ability to isolate and select new phages rapidly and cost‐effectively positions this “living medicine” as a sustainable and targeted alternative to restore the ecological balance required for durable diabetic tissue repair [142].

### 5.4 Combining Phages With Probiotics/Consortia

The combination of bacteriophages and probiotic consortia is a sophisticated sequential therapeutic strategy designed to transform the dysbiotic diabetic wound environment into a stable pro‐healing ecosystem. In this “debulking” phase, bacteriophages serve as precise ecological tools, specifically lysing dominant MDR pathogens such as *Staphylococcus aureus* and *Pseudomonas aeruginosa* while sparing the commensal microbiota [13, 127, 143]. By dispersing the EPS matrix and reducing the absolute bacterial burden, phages end the local hyperinflammatory response and open metabolic and spatial niches previously occupied by pathogenic biofilms [144, 145, 146]. The subsequent probiotic recolonization phase uses these vacated niches to establish beneficial strains, such as *Bifidobacterium* and *Lactobacillus*, which prevent pathogen rebound through competitive exclusion and the permanent occupation of critical adhesion sites [72].

These antagonistic microbes stabilize the newly formed community by continuously secreting organic acids, bacteriocins, and quorum‐sensing inhibitors, ensuring that any surviving pathogen remains in a non‐virulent state [88]. This synergy also extends to host modulation, as probiotics are safely engrafted in a pathogen‐depleted environment, providing signals that drive macrophage polarization toward the reparative M2 phenotype and stimulate neovascularization through the release of bioactive EVs [147]. By combining the rapid lytic efficacy of phages with the long‐term metabolic and immunomodulatory benefits of designed probiotic consortia, this approach achieves a multilayered defense that not only resolves treatment‐resistant infections but also restores the biological signaling required for durable diabetic tissue repair [148].

## 6 Microbial Antagonism, Angiogenesis, and Epithelial Repair: Mechanistic Links

### 6.1 Angiogenesis and Vascular Remodeling

The orchestration of angiogenesis and vascular remodeling through microbial antagonism represents a transformative mechanistic link in diabetic wound repair, as beneficial microorganisms actively counter the ischemic and hyperinflammatory barriers that impede neovascularization. Evidence from preclinical and clinical studies confirms that the topical application of certain probiotics, including *Lactiplantibacillus plantarum*, *Lacticaseibacillus casei*, and *Lacticaseibacillus rhamnosus GG*, significantly enhances vascular density and endothelial function in the diabetic microenvironment [70, 93, 94]. For example, *Lacticaseibacillus rhamnosus GG* and *Lacticaseibacillus casei* promote rapid tissue regeneration by releasing EVs that act as precise delivery systems for signaling molecules, such as miR‐21‐5p [70]. These vesicles are internalized by vascular endothelial cells and keratinocytes, directly stimulating their proliferation and migration to initiate new capillary network formation and to restore the epithelial barrier [7]. Clinical studies using probiotic soybean‐based concentrates containing *Lactiplantibacillus plantarum*, *Lactobacillus acidophilus*, and *Lacticaseibacillus casei* have shown similar results, with 83% of non‐infected DFUs achieving complete closure, primarily owing to improved angiogenesis and fibroplasia [84, 95].

The role of microbial metabolites and host mediators is central to this regenerative process, and VEGF is a primary target for microbiome‐based interventions. Probiotics and their associated AMPs, such as LL‐37, have been shown to upregulate the expression of VEGF‐A and TGF‐β, resolve ischemic stalling and stimulate granulation tissue formation [82, 149, 150, 151]. This is often achieved by facilitating a phenotypic switch in host macrophages, driving them from a pro‐inflammatory M1 state to a reparative M2 phenotype, which then serves as a powerhouse for VEGF and PDGF secretions [152]. Additionally, innovative strategies targeting hypoxia‐inducible factor (HIF) pathways, such as nanotherapeutics to stabilize HIF‐1α, have proven successful in boosting VEGF and FGF‐2 levels in hypoxic diabetic tissues, thereby accelerating neovascularization [153, 154].

Restoring nitric oxide (NO) bioavailability is another critical aspect of vascular remodeling mediated by probiotics. In patients with DFU, probiotic supplementation has been associated with significantly increased plasma NO concentrations, which are essential for maintaining normal endothelial vasodilation and tissue perfusion in compromised diabetic limbs [92, 155, 156, 157]. Meanwhile, antagonistic microbes deploy a potent antioxidant arsenal to neutralize ROS, which would otherwise induce endothelial senescence and degrade the ECM scaffold [158, 159]. Some probiotics produce antioxidant enzymes such as SOD and catalase as well as metabolites, such as glutathione and folate, enabling ROS scavenging and protection against oxidative stress, including mitochondrial impairment [160, 161, 162]. This reduction in oxidative stress preserves the structural integrity of newly formed vessels and allows the wound to transition smoothly from a chronic inflammatory state to active epithelial repair and neovascularization [163, 164].

### 6.2 Epithelial Regeneration and Matrix Remodeling

**FIGURE 4:**
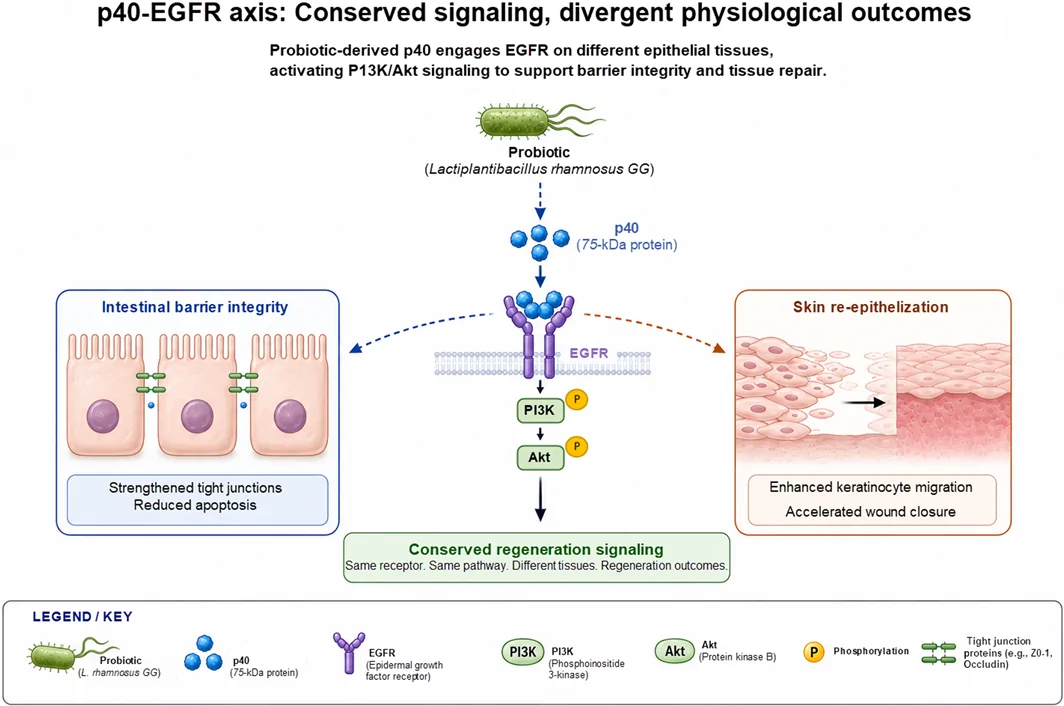
Proposed mechanistic framework linking microbial signaling pathways to epithelial regeneration and tissue repair. A conserved signaling bridge. This schematic illustrates the cross‐tissue conservation of repair mechanisms triggered by beneficial microbes. Probiotic‐derived proteins, specifically p40 (secreted by *Lacticaseibacillus rhamnosus* GG), serve as exogenous ligands for the host Epidermal Growth Factor Receptor (EGFR). Activation of the conserved PI3K/Akt signaling axis facilitates divergent but functionally synergistic outcomes: promoting barrier integrity and anti‐apoptosis in the intestinal epithelium (top), while overcoming the cellular senescence of the diabetic wound to stimulate keratinocyte migration and proliferation (bottom). This framework justifies the translation of gut‐derived microbial insights into novel strategies for chronic wound restoration. The illustrated pathways represent a conceptual integration of findings from multiple experimental systems and highlight putative conserved mechanisms relevant to diabetic wound healing.

Epithelial regeneration and matrix remodeling in chronic wounds involve microbial signals that affect the redox balance, growth factor release, and stem cell activation to restore barrier integrity. Although a definitive, unified multi‐tissue biological principle is still being established, emerging evidence suggests that a conserved framework governs these interactions. Identifying these commonalities is essential for developing a regenerative microbiological approach that is applicable to different chronic wound phenotypes [165]. Data from intestinal and mucosal models showed that commensal microbes are central regulators of epithelial restitution. For example, the probiotic *Lacticaseibacillus rhamnosus GG* secretes the functional protein p40, which stimulates epithelial cell proliferation by activating the epidermal growth factor receptor (EGFR) and PI3K/Akt signaling pathway [166, 167]. Microbial signaling uses a conserved p40/EGFR/PI3K axis to bypass host cell senescence and to initiate repair (mapped as a cross‐tissue bridge in Figure 4). In the gut, metabolites such as SCFAs and prebiotics such as inulin promote the differentiation of Lgr5+ stem cells into mature epithelial cells, while upregulating tight junction proteins such as ZO‐1 and occludin [168]. Furthermore, microbe‐induced ROS signaling plays a dual role: while excessive oxidative stress in diabetic tissue is destructive, the controlled metabolic activity of beneficial microbes helps maintain a balanced redox state necessary for cellular signaling [169]. Probiotics reinforce this balance by releasing antioxidant enzymes such as SOD and catalase to scavenge ROS, thereby preventing mitochondrial collapse and protecting newly formed tissues from oxidative damage [170].

These conserved mechanisms are highly relevant to the diabetic skin microenvironment, where keratinocyte migration and proliferation are severely impaired by chronic hyperglycemia, ischemia, and persistent inflammation [171, 172]. In DFUs, pathogenic biofilms and the accumulation of AGEs induce premature cellular senescence in fibroblasts and keratinocytes, limiting their proliferative capacity and trapping the wound in a non‐healing state [173, 174]. Beneficial microbes and their derived AMPs can overcome these biological barriers. For example, the peptide AMP‐IBP5 counteracts the inhibitory effects of high glucose on keratinocytes by activating signaling pathways involving EGFR, STAT3, and MAPKs [175, 176]. Additionally, probiotic strains, such as *Lacticaseibacillus rhamnosus GG* and *Lacticaseibacillus casei* release EVs containing miR‐21‐5p, which are internalized by vascular endothelial cells and keratinocytes to directly stimulate the migration and proliferation required for effective re‐epithelialization [70] (Figure 4, Skin re‐epithelialization endpoint).

Restoring the ECM is a major challenge in diabetic tissue repair and is often hindered by the overexpression of matrix metalloproteinases (MMPs), particularly MMP‐9 [177, 178]. This excessive protease activity, driven by dysbiotic microbial triggers, leads to the abnormal degradation of newly formed ECM components and inactivation of essential growth factors [179]. Emerging “regenerative microbiology” strategies employ layer‐by‐layer (LbL) technology to deliver siRNA that suppresses MMP‐9 expression directly in the wound bed, promoting ECM accumulation and accelerating wound closure [42]. Beneficial microbes, such as *Lactiplantibacillus plantarum* further support this remodeling phase by increasing TGF‐β levels and modulating collagen deposition, ensuring the formation of organized tissue structures rather than the disordered bundles observed in hypertrophic scarring [73]. By combining infection control with active stimulation of progenitor cells and fibroblast function, these microbiome‐based interventions offer a targeted approach to restore the functional and structural integrity of the diabetic skin [7].

### 6.3 Translational Logistics: Economics, Stability, and Clinical Feasibility

The transition from traditional wound dressings (such as moisture‐retaining hydrogels, alginates, and silver‐infused foams) to engineered microbial consortia and live biotherapeutic products (LBPs) presents distinct economic and logistical challenges [180]. Traditional advanced dressings are cost‐effective due to their long room‐temperature shelf lives (typically 2–5 years) and straightforward supply chains. In contrast, biological agents require complex manufacturing and handling processes. Scaled production of live multi‐strain consortia demands precise bioreactor controls to maintain intended strain ratios, increasing initial production costs compared to synthetic materials. Clinically, the viability of these biological therapeutics depends on unbroken cold‐chain logistics (typically stored at 2°C–8°C, or deep freezing at −80°C for certain liquid formulations). To enhance stability and extend shelf life to commercially viable periods, lyophilization with protective lyoprotectants (such as trehalose) is widely used, allowing reconstitution immediately before application [181, 182]. In clinical practice, these agents are applied using simple protocols, such as pre‐measured topical sprays, hydrogel mixtures, or pre‐impregnated stable matrices, ensuring precise dosing without placing undue burden on healthcare providers.

### 6.4 Immune Modulation and Resolution of Chronic Inflammation

Resolving chronic inflammation in diabetic wounds requires a fundamental shift from persistent neutrophil‐dominated injury to a balanced, reparative immune profile. In the dysbiotic diabetic microenvironment, excessive and unregulated neutrophil infiltration serves as a biomarker for non‐healing, as these cells release destructive proteases, such as elastases and ROS, sustaining a self‐perpetuating cycle of tissue damage [183, 184, 185]. Microbial antagonists, particularly probiotic strains such as *Lactiplantibacillus plantarum* and *L*. *bulgaricus*, actively intervene in this cycle by suppressing pro‐inflammatory mediators and inducing macrophage polarization from the destructive M1 phenotype to the reparative M2 phenotype [186, 187]. This transition is critical because M2 macrophages resolve the hyperinflammatory state by secreting anti‐inflammatory cytokines, such as IL‐10, and essential growth factors, such as TGF‐β, which are necessary for advancing the wound into the proliferative phase. Specific AMPs, such as LL‐37 and human *β*‐defensins, bridge innate and adaptive immunity by modulating T‐cell homeostasis and promoting the differentiation of Th17 cells, which assists in long‐term pathogen clearance and barrier restoration [91, 152].

Immune reprogramming is closely linked to pathogen suppression, which creates conditions that support tissue repair. Microbial antagonists eliminate the main triggers for sustained TLR4 and NF‐κB signaling by reducing the absolute bacterial burden and dispersing pathogenic biofilms, thereby decreasing the expression of pro‐inflammatory cytokines, such as TNF‐α and IL‐1β [94, 188]. This dual effect ensures that as the infection resolves, the host's innate responses are directed toward regeneration rather than defense, preventing collateral damage typically associated with chronic neutrophil activation and NETosis [80, 189]. Metabolic products from these beneficial microbes, such as SCFAs like butyrate, further stabilize this environment by inhibiting histone deacetylases (HDACs), which downregulate inflammatory gene expression in macrophages and promote a tolerogenic state [190, 191]. Precision ecological tools, such as bacteriophages, enhance this process by selectively lysing dominant pathogens and ending the inflammatory stimulus while sparing commensals that support adaptive immune signaling [192, 193]. Ultimately, the synergy between direct microbial antagonism and restoration of a balanced M1/M2 profile resolves inflammatory arrest, enabling smooth progression toward granulation, angiogenesis, and functional tissue restoration. Lessons from liver regeneration models further indicated that a balanced microbiome can train host innate responses, shifting them from chronic inflammation to metabolic repair. This is exemplified by the production of enterically derived high‐density lipoprotein (HDL3), which prevents the inflammatory activation of macrophages by binding to LPS‐binding proteins and shielding tissue from further injury [194].

## 7 Translational Barriers and Design Challenges

### 7.1 Strain Variability and Context Dependence

The clinical translation of microbiome‐based interventions for chronic diabetic wounds faces significant barriers and design challenges, primarily because of the inherent complexity of biological systems and the hostile diabetic microenvironment [165]. A major obstacle is strain variability, as therapeutic effects are highly strain‐specific and cannot be generalized across species. For example, although various strains of *Lactiplantibacillus plantarum* have been widely studied for their antagonistic properties, research comparing *Lactobacillus acidophilus KLDS1*.*1003* and *KLDS1*.*0901* has shown that *Lactobacillus acidophilus* is significantly more effective at reshaping the microbiota and improving metabolic markers [195, 196]. This strain‐level divergence also affects surface hydrophobicity, stress tolerance, and specific matrix interactions, making the selection of a universal probiotic species problematic [197, 198]. Additionally, reliance on multi‐strain consortia in clinical trials can obscure the individual dosing and viability requirements of specific microbes, complicating regulatory approval and standardized manufacturing [198, 199].

**FIGURE 5:**
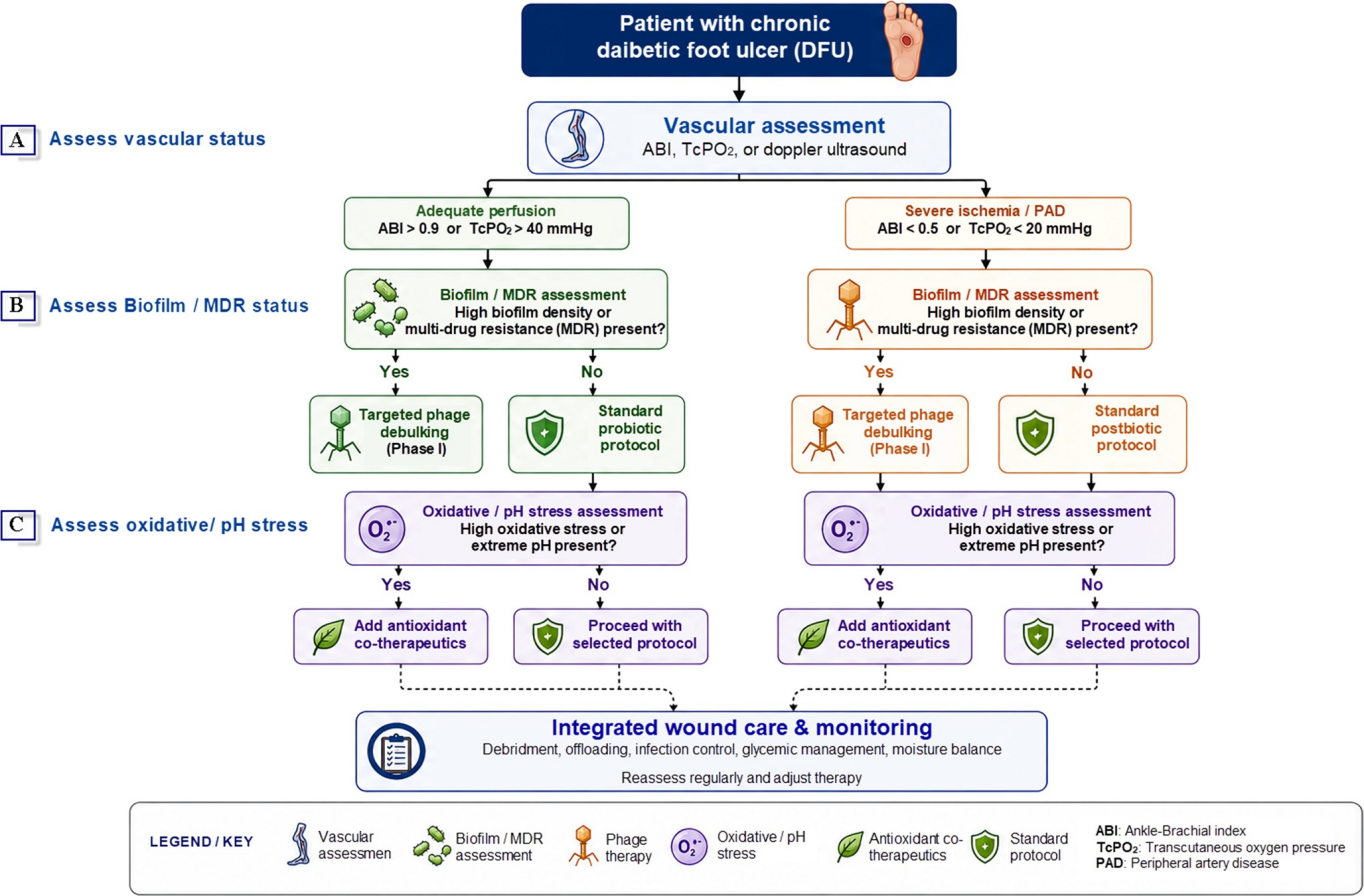
Proposed precision‐stratification framework for microbiome‐based interventions in diabetic wounds. (A) Vascular assessment: vascular status serves as the primary clinical filter. Patients with adequate distal perfusion (ABI > 0.9 or TcPO_2_ > 40 mmHg) may be considered for live probiotic consortia, whereas patients with severe ischemia or peripheral arterial disease (ABI < 0.5 or TcPO_2_ < 20 mmHg) may require metabolically independent postbiotics or extracellular vesicles. (B) Biofilm/MDR assessment: secondary stratification according to biofilm density or the presence of multidrug‐resistant pathogens guides the use of initial targeted phage‐mediated debulking. (C) Oxidative/pH stress assessment: evaluation of oxidative stress and wound pH informs the potential addition of antioxidant co‐therapeutics before proceeding with integrated wound care and monitoring. These cell‐free agents and adjunctive strategies are intended to support regenerative effects under conditions that may compromise the viability of live microorganisms. This framework illustrates potential patient‐stratification strategies based on current evidence and has not been prospectively validated for clinical decision‐making.

The context‐dependence of these therapies is further influenced by numerous host factors and the wound microenvironment, both of which can profoundly affect therapeutic success. DFUs are highly heterogeneous, including neuropathic, ischemic, and neuroischemic phenotypes that exhibit varying degrees of vascular compromise and tissue hypoxia [200, 201]. The prevalence of peripheral arterial disease (PAD) in approximately 50% of DFU patients introduces a significant physiological barrier and severe local hypoxia [202]. Although many beneficial *Lactobacillus* and *Bifidobacterium* species are microaerophilic or anaerobic, the extremely oxygen‐depleted and nutrient‐poor environment of an ischemic wound may impair their metabolic activity and long‐term viability. This raises the question of the relative translational utility of postbiotics, defined as cell‐free supernatants or purified microbial metabolites (e.g., SCFAs, bacteriocins, and exopolysaccharides) compared to live biotherapeutics. Postbiotics offer a distinct advantage in ischemic settings as they do not require a viable niche to exert their effects. Furthermore, postbiotics eliminate the risks associated with bacterial translocation in immunocompromised patients and offer greater stability than topical formulations do. However, although postbiotics provide a stable, fixed dose of therapeutic molecules, they lack the dynamic, self‐regulating feedback loops of live consortia that can sense and respond to a changing wound microenvironment. Therefore, in cases of severe ischemia, a postbiotic‐first approach may be more viable, whereas live biotherapeutics may be reserved for patients with an adequate distal perfusion (Figure 5A, adequate distal perfusion branch). Clinical intervention must therefore be stratified by vascular status, opting for postbiotics in cases of severe ischemia where live microbial viability is compromised (Figure 5A, severe ischemia/PAD pathway). Additionally, the alkaline shift in pH typical of chronic diabetic wounds can destabilize probiotic formulations and favor the persistence of opportunistic pathogens [203] (Figure 5C).

The effectiveness of “microbes against microbes” also depends on the host's glycemic control and immune status of the host, as chronic hyperglycemia induces oxidative stress and impairs the innate immune responses required to cooperate with probiotic signals [200, 204, 205]. Patients often have systemic comorbidities such as chronic kidney disease (CKD), which increases clinical complexity and worsens overall healing trajectories [206, 207]. The existing polymicrobial biofilm also acts as a significant barrier; interactions between administered therapeutic strains and established resistant microbial communities remain insufficiently characterized, presenting a risk of unpredictable ecological outcomes [73, 208] (Figure 5B). Current clinical management often includes systemic antibiotics or topical antiseptics, such as iodine or silver‐based agents, which can inadvertently suppress the activity of therapeutic probiotics and disrupt the intended restorative shift in the wound microbiome [209, 210]. These factors underscore the need for a precision medicine approach that can tailor microbiome‐based treatments to the specific microbial profile and physiological state of each patient.

A major obstacle in translating these therapies from bench to bedside is that standard rodent models often fail to accurately predict human wound response. This translational gap results from key differences in anatomy and healing physiology. Rodent skin is loosely attached to underlying structures and contains the panniculus carnosus muscle layer, which drives wound healing primarily through rapid tissue contraction. In contrast, human skin lacks this muscle layer and relies on re‐epithelialization and granulation tissue formation, a much slower process driven by lateral cell migration. Additionally, skin tension dynamics differ fundamentally: rodents experience low tension across loose skin, while human extremities experience high lateral skin tension that alters local mechanotransduction and healing rates [211, 212]. The local microenvironment also differs significantly in microbiome density and composition. Specific‐pathogen‐free (SPF) laboratory rodents live in highly controlled environments and have a relatively low‐density, less complex skin microbiome, whereas human DFUs typically harbor dense, polymicrobial communities and complex biofilms composed of diverse aerobes and anaerobes [77, 213, 214].

### 7.2 Delivery Systems and Matrices

The clinical success of microbiome‐based interventions relies on the rational design of next‐generation delivery platforms capable of navigating the hostile and heterogeneous environment of diabetic wounds. A major challenge in formulation is selecting appropriate matrices, such as topical PEG‐glycerol gels, oleogels, and hydrogels, which must preserve microbial viability and ensure controlled release [215, 216]. Conventional microencapsulation techniques, including spray drying and lyophilization, enhance strain resistance to environmental stress but often face scalability constraints and can produce irregularly shaped, porous particles that offer inadequate protection [217, 218]. Advanced stimuli‐responsive hydrogels provide a more sophisticated approach, as they can be engineered to adjust their properties or release therapeutic payloads in response to local triggers, such as pH shifts, temperature changes, or glucose levels [219]. For example, programmable hierarchical hydrogel dressings enable the sequential release of multiple agents, which is essential to address the evolving needs of chronic wounds as they progress through different healing stages [220].

Integrating these therapies into clinical practice requires precise timing relative to standard‐of‐care procedures, specifically debridement and antibiotic regimens. Clinical protocols often specify that probiotic application should follow surgical debridement to ensure that beneficial microbes are delivered to a viable wound bed [15]. However, the concurrent use of systemic antibiotics or topical antiseptics, such as silver or iodine, presents a significant design challenge, as these agents may inadvertently suppress applied therapeutic strains [221]. To address this, researchers are exploring bioengineered dressings and nanofiber scaffolds that serve as protective barriers, shielding probiotics from external antimicrobial agents while facilitating their gradual engraftment into the wound [222, 223].

Effective dosing strategies must also address these environmental barriers, with typical oral doses ranging from 10^7^ to 10^12^ CFU per day. Topical applications in clinical trials have used concentrations of approximately 2 × 10^9^ CFU per gram to achieve measurable outcomes [224, 225]. Achieving rapid metabolic resuscitation upon release is essential for therapeutic efficacy; however, viability loss during drying and storage of live formulations remains a major industrial limitation [226, 227, 228]. Consequently, there is a growing shift toward postbiotic formulations that use cell‐free supernatants or microbial metabolites, which may offer a more stable, controllable, and safer alternative for high‐risk, heavily colonized diabetic populations, where the risk of live bacterial translocation is a concern. The transition from experimental adjuncts to routine clinical tools will ultimately require multimodal platforms that combine targeted delivery with real‐time monitoring using integrated biosensors to tailor the dose to the specific microbial and physiological landscape of the wound.

### 7.3 Biosafety, Containment, and Biosecurity

The clinical implementation of microbiome‐based interventions in a chronic diabetic wound environment involves complex safety and immune considerations, as the introduction of live biological agents into compromised tissues carries inherent risks. A primary concern for high‐risk populations, such as those with DFUs, is the risk of bacteremia and systemic translocation, where administered live probiotics may cross an impaired biological barrier and enter the bloodstream, potentially leading to sepsis. Although many clinical trials have reported acceptable short‐term tolerability, these studies are often underpowered to detect rare but clinically significant systemic infections in patients with multiple comorbidities such as CKD [229]. Additionally, both live microbes and bacteriophages can trigger excessive inflammation; specifically, the rapid destruction of pathogens by phages releases large quantities of endotoxins, which can induce a Jarisch–Herxheimer reaction characterized by chills, fever, and systemic inflammatory responses [230]. Initial microbial transplantation has also been observed to cause a temporary inflammatory response in the host before ecological stabilization is achieved [73].

Beyond immediate inflammatory events, the potential for horizontal gene transfer (HGT) and exchange of mobile genetic elements presents a significant safety concern. Probiotic strains and bacteriophages can act as vectors for transmitting antibiotic‐resistance genes or virulence factors through plasmids and prophages [231]. In particular, generalized transduction allows phages to mistakenly package host bacterial DNA into their capsids, thereby facilitating the spread of pathogenicity across the wound microbiome. Although bacteriophages are engineered for high host specificity to preserve beneficial commensal communities, safety evaluations must consider the potential for secondary dysbiosis. This risk arises not from off‐target killing but from broader ecological shifts within the wound microbiome following the rapid lysis of dominant pathogens. Additionally, the theoretical potential for HGT, in which phages may inadvertently facilitate the spread of virulence factors or antibiotic resistance genes, represents a distinct regulatory challenge that differentiates the intended precision of phage therapy from its long‐term ecological risks [137, 232].

The host immune response to therapeutic phages and live microbes further affects their safety and efficacy. Administered phages are recognized as foreign by the innate immune system through pattern recognition receptors (PRRs), such as toll‐like receptors (TLRs) on macrophages and keratinocytes. This recognition activates macrophages, resulting in the phagocytosis of therapeutic phages and the secretion of pro‐inflammatory cytokines, which may neutralize the therapy if not properly managed [119, 231, 233]. Evidence has also shown that repeated or high‐dose administration can induce the production of phage‐specific antibodies, potentially limiting the effectiveness of the same phage in future treatments [234].

To address these complex risks, regulatory expectations for clinical translation have increased, requiring clinical‐grade manufacturing under strict Good Manufacturing Practice (GMP) standards. Comprehensive characterization is now essential, with regulatory bodies such as the EFSA increasingly mandating whole‐genome sequencing to confirm the absence of antibiotic resistance genes or toxins in probiotic strains [180, 235]. For bacteriophage therapy, thorough genomic analysis is required to exclude temperate or lysogenic phages that can integrate their DNA into the host genome instead of inducing beneficial bacterial lysis. Quality control protocols must also prioritize the purification of phage lysates to remove bacterial debris and residual growth media, which can trigger adverse immune reactions. The transition from experimental adjuncts to mainstream medicine ultimately requires harmonized testing standards, robust safety endpoints, and a precision medicine approach tailored to each patient's unique microbial and immune profiles.

Introducing high‐density microbial therapies into the active resistome of a chronic wound raises significant biosafety concerns regarding HGT. Therapeutic strains may inadvertently transfer mobile genetic elements to resident opportunistic pathogens, such as *Staphylococcus aureus*, or acquire resistance elements from them. To mitigate this risk, researchers have incorporated multilayered genetic safeguards into clinical‐grade strains. Chief among these is engineered metabolic auxotrophy, in which therapeutic bacteria are modified so they cannot synthesize an essential nutrient, such as specific amino acids like D‐alanine or thymidine [236]. As survival depends on controlled nutrient availability, auxotrophic strains can be designed to persist only within a restricted therapeutic window. Synthetic biology approaches integrate dual‐stage, unlinked biocontainment systems, including toxin‐antitoxin kill switches that trigger genomic self‐destruction if the bacteria detect an environmental change or escape from the targeted wound site, ensuring they cannot persist in the patient or spread into the environment [237].

### 7.4 Regulatory and Ethical Landscape

The clinical integration of LBPs and bacteriophage therapy is currently navigating a fragmented global regulatory and ethical landscape, as traditional pharmaceutical frameworks struggle to accommodate “living medicines.” [156, 238]. The use of these therapies varies significantly across jurisdictions. In the United States, the Food and Drug Administration (FDA) classifies bacteriophages as drugs or biologics, requiring them to meet rigorous medicinal product licensing requirements, including good manufacturing practices (GMP) and multi‐phase clinical trials [180]. Although the first US clinical trial for intravenous phage therapy was approved in 2019, many interventions still occur under the emergency investigational new drug (eIND) protocol [231]. For probiotics, the FDA relies on CFU‐based labeling, although enforcement remains inconsistent compared with other regions.

In the European Union, regulation is often inconsistent, as member states have the freedom to regulate certain procedures such as FMT, at the national level [239, 240]. Belgium has pioneered a “magistral preparation” pathway, allowing phages to be prepared in compounding pharmacies for individual patients, bypassing some constraints of large‐scale industrial manufacturing [241]. Poland facilitated phage therapy through an “experimental treatment” framework at the Ludwik Hirszfeld Institute under the Helsinki Declaration [242]. In contrast, Russia and Georgia have a long‐standing history in which phage cocktails are widely available in pharmacies as standard care. In Asia, Food Safety and Standards Authority of India (FSSAI) requires detailed strain‐level documentation for probiotics, whereas China has established two pathways: one for fixed‐ingredient products and another for personalized treatments through investigator‐initiated trials.

A fundamental challenge is standardizing these “ecological” therapies within a regulatory system designed for single‐molecule drugs [243]. Unlike static chemical compounds, phages and live microbes are self‐replicating, evolving entities with high strain specificity; therefore, a single predefined preparation may have a short useful lifespan as resistance emerges [244]. Traditional pharmaceutical models prioritize large‐scale distribution and lack customization, which directly conflicts with the need for personalized and timely adaptation of microbial consortia to a patient's specific pathogen profile. This mismatch makes regulatory testing for safety and efficacy much more complex and expensive.

Furthermore, the ethical considerations surrounding these therapies remain significant. Regulatory gray areas exist for agents, such as AMPs, that function as both antimicrobials and immunomodulators, complicating their classification. Ethical dilemmas also arise regarding informed consent and patient selection, particularly when potent antimicrobial agents are introduced into destabilized ecosystems. Additionally, the lack of standardized sampling, sequencing, and data analysis protocols across research groups has led to inconsistent outcomes, undermining the predictive validity required for broad clinical adoption. Ultimately, the transition to microbiome‐guided precision medicine requires a shift toward harmonized international guidelines and flexible regulatory frameworks that recognize the unique dynamic nature of ecological interventions.

Despite promising advances, the evidence supporting microbiome‐based interventions for diabetic wound repair remains largely preclinical, with many mechanistic insights derived from in vitro studies and animal models. Therapeutic effects are often strain‐specific and context‐dependent, limiting broad generalization across microbial species, formulations, and patient populations. Therefore, well‐designed, adequately powered randomized controlled trials are needed to establish efficacy, optimize treatment protocols, and define long‐term safety and clinical applicability.

## 8 Future Perspectives and Clinical Translation Pathways

Although the conceptual foundation of regenerative microbiology is strong, translating these therapies into routine clinical care will depend on overcoming several key technological and regulatory challenges.

➢ **Precision multi‐omics screening**: Future research should prioritize high‐throughput metagenomic and metabolomic profiling of patient wound beds to match specific live biotherapeutic consortia to each patient's local resistome and pathogen profile.
➢ **Advanced delivery matrices:** A major bioengineering priority is the development of responsive smart hydrogels that shield live therapeutic strains from environmental stresses (e.g., high glucose levels and alkaline pH) while enabling controlled, sequential release of postbiotics and phages over time.
➢ **Regulatory standards and clinical evidence:** Clear regulatory pathways for LBPs must be established with agencies such as the FDA, including strict metrics for batch‐to‐batch manufacturing consistency, stability, and standardized safety testing to minimize the risk of systemic translocation or gene transfer. Parallel efforts should focus on multicenter, randomized controlled trials to evaluate long‐term safety, healing rates, and cost‐effectiveness relative to current standard‐of‐care.

## 9 Conclusions and Summary Insights

This review introduces the emerging paradigm of Regenerative Microbiology, a strategy that has shifted from non‐specific antimicrobial elimination to the targeted use of microbial ecology to heal chronic diabetic wounds. By integrating competitive exclusion, targeted phage‐mediated biofilm degradation, and localized signaling modulation, this framework addresses the root causes of non‐healing ulcers, including persistent inflammation, biofilm defense networks, and impaired tissue repair. Transitioning from single‐strain treatments to coordinated microbial consortia offers a promising approach to restore balanced, healing‐supportive ecosystems in chronic wounds, and provides a sophisticated alternative to traditional dressings.

## Author Contributions

S.S.S. conceived and supervised the study and wrote the manuscript.

## Ethics Statement

The author has nothing to report.

## Consent

The author has nothing to report.

## Conflicts of Interest

The author declares no conflicts of interest.

## Data Availability

Data sharing not applicable to this article as no datasets were generated or analyzed during the current study.
